# Medical genetics and genomic medicine in India: current status and opportunities ahead

**DOI:** 10.1002/mgg3.150

**Published:** 2015-05-14

**Authors:** Shagun Aggarwal, Shubha R Phadke

**Affiliations:** 1 Department of Medical Genetics, Nizam's Institute of Medical SciencesHyderabad, India; 2 Diagnostics Division, Centre for DNA Fingerprinting and DiagnosticsHyderabad, India; 3 Department of Medical Genetics, Sanjay Gandhi Postgraduate Institute of Medical SciencesLucknow, India

## Demographic Features of India

**Figure 1:**
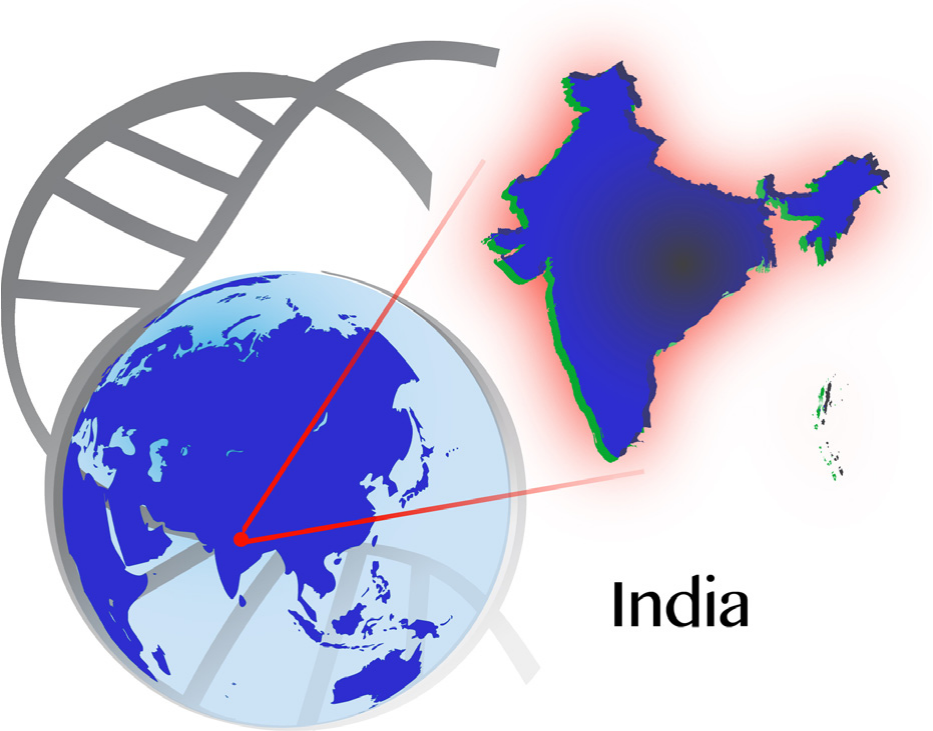

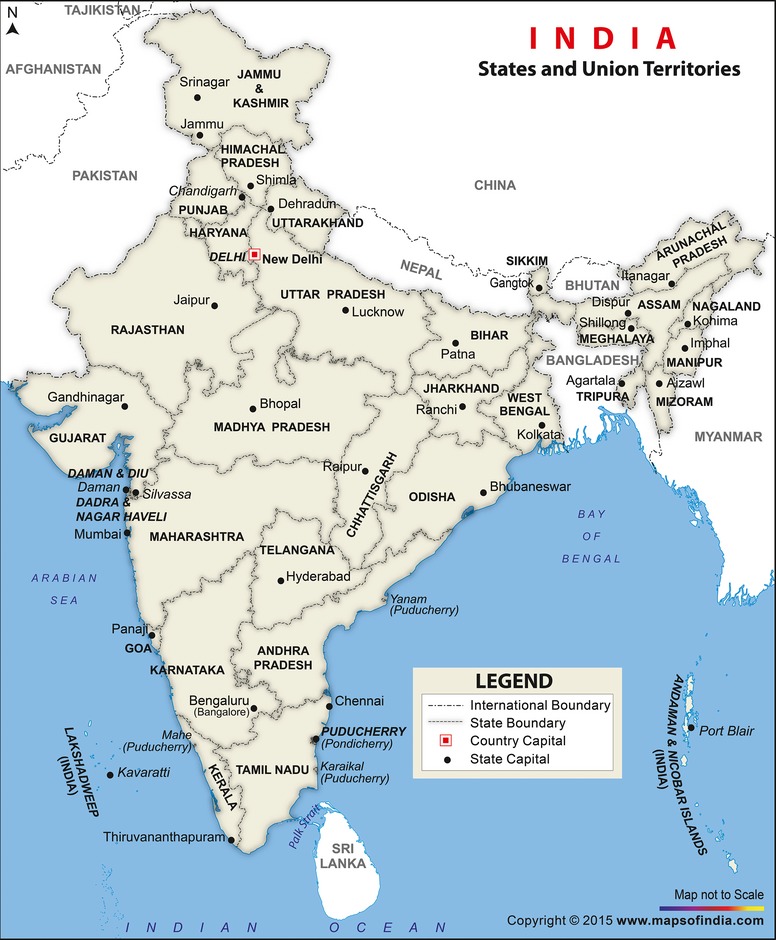
Political Map of India.

India is the sixth largest country of the world in size and with a population 1.21 billion (http://www.censusindia.gov.in) has the distinction of being the second most populous country of the world, housing 17.5% of all humans (http://www.prb.org). It is part of the Indian subcontinent that is comprised of the surrounding countries of Pakistan, Bangladesh, Srilanka, Nepal, and China with whom it shares common cultural and anthropological roots. The southern part of India is the Indian peninsula surrounded by the Indian Ocean, the Bay of Bengal, and the Arabian Sea (Fig. 1). The Indian subcontinent has been the seat of some of the oldest civilizations of the world with the earliest historically documented remains dating back to as early as 70,000 years. This region is believed to have been initially habituated 55,000–80,000 years back by migration from the African continent as indicated by mitochondrial and Y chromosome DNA genotypes (Majumdar 2010; Tamang et al. 2012). Subsequently, multiple events of migration and invasions from northwestern and eastern sides led to population admixture giving rise to highly heterogeneous population groups in this country. Presently, there are believed to be four main ethno-racial groups- the Caucasoids, Australoids, Mongoloids, and Negritos- in India. The Caucasoids inhabit the northern and northwestern part and speak the Indo-Aryan langauages, the Australoids the southern part and speak the Dravidian languages and the Mongoloids the northeastern part of the country and speak the Tibeto-Burman languages. The Negritos are confined to the Andaman & Nicobar islands which lie in the extreme southeastern part of the country. The majority of the modern Indian population is an admixture of two large genetically divergent and heterogeneous population groups that mixed in ancient times (about 1200–3500 bc), known as Ancestral North Indians (ANI) or the Caucasoids and Ancestral South Indians (ASI) or the Australoids. Overall there are more than 4000 anthropologically distinct groups and 22 languages with various dialects in this diverse nation (Majumdar 2010; Narang et al. 2010; Tamang et al. 2012). Genetic studies for classifying the population into subgroups and identifying the origins have been at the best incomplete due to the vast population and extreme complexity. This poses challenges for genetics and genomic medicine research. In recent years, the Indian Genome variation consortium project has studied polymorphisms in 900 genes from 55 different population groups of India and these variations have been catalogued in the Indian Genome Variation browser. This forms an important database for design of further studies of multifactorial as well as single gene disorders (Narang et al. 2010). The geographical & linguistic disparity is further complicated by the caste system and religious boundaries, which are associated with high degree of endogamy, although recent reports have indicated some degree of admixture (Tripathi et al. 2008; Majumdar 2010; Tamang et al. 2012; Juyal et al. 2014). Consanguinity is as high as 20–30% in some specific populations, indicating the possibility of clusters of specific diseases and founder mutations (Bittles 2002; Juyal et al. 2014). Although almost all known genetic disorders have been reported in India, and regional distribution for many conditions historically known, prevalence data and the mutation profiles are not well known for the majority. The underlying population genetic heterogeneity poses significant challenges in this regard. In addition to the complexity and the magnitude of population, the presence of disparity in economical and infrastructural resources at the level of population subgroups as well as individuals further complicates the health care delivery and access to genetic services (Balarajan et al. 2011).

## Health Services in India

The health services in India are available as a public service which is provided by the Government of India under the Ministry of Health and Family Welfare as well as a private service which is provided by various corporate organizations in addition to individual practices (Balarajan et al. 2011, http://www.mohfw.nic.in). India spends 1.2% of its GDP on health, and provides completely free or highly subsidized health services to the economically deprived groups through the public health system (Balarajan et al. 2011). This system consists of tertiary level hospitals in the larger cities, secondary level hospitals which are the district and taluka hospitals and the primary health centers for both the urban and rural population. The tertiary level hospitals are usually allied to a medical school and are involved in training of doctors and other medical personnel. A large proportion of clinical as well basic scientific research is conducted under the aegis of these institutions many of which like the All India Institute of Medical Sciences, New Delhi; Post Graduate Institute of Medical Education & Research, Chandigarh; Sanjay Gandhi Post Graduate Institute of Medical Sciences, Lucknow, etc. are recognized for clinical excellence as well as research contributions (http://www.mohfw.nic.in). The planning of health service delivery, training of personnel, and allocation of budget is governed by the Ministry of Health & Family Welfare under the Central government. However, the execution of the health system delivery is primarily governed by the respective state governments of the 36 different states of India (http://www.mohfw.nic.in). Insufficiency of budgetary allocations leads to numerous gaps in service delivery, and only 25–30% of the population is actually able to access this system (Balarajan et al. 2011; Jindal 2014). Hence, the private health services cater to the health needs of the majority of the population (Balarajan et al. 2011; Jindal 2014). This system is highly heterogeneous and its pattern differs across different regions of the country. Larger urban areas have super-specialty hospitals governed by various corporate bodies and these are primarily accessed by the economically privileged class. Many of them offer state of the art medical care and are popular destinations for medical tourism from across the world. However, smaller nursing home practices and individual practices are responsible for providing health care to the majority of Indians both in rural and urban areas. These in turn are varied in their level of care depending on the expertise of the doctors and the infrastructure availability (Balarajan et al. 2011; Jindal 2014). Most medical care is funded by individuals themselves and in some cases by the employer while private medical insurance is taking roots in India (Balarajan et al. 2011; Jindal 2014). The quality regulation of medical practice is primarily the responsibility of the Medical Council of India (MCI) which lays down guidelines for both the training of the doctors as well as their professional conduct (http://www.mciinidia.org). The National Board of Examinations is another governing body which is involved in training of doctors in addition to MCI approved courses in medical institutions (http://www.natboard.edu.in).

## Genetic Services

With decreasing trend of infant mortality rates in India, genetic disorders are emerging as an important group of medical ailments requiring attention at a priority level. Genetic services in India can be dated back to the commencement of small genetic units in few premier cities like Delhi, Lucknow, Bangalore, Mumbai, and Pune as multicentric research programs for prevention of neural tube defects, etiological diagnosis of mental retardation and high-resolution cytogenetics funded by the Indian Council of Medical Research(ICMR) in the late 1980s (www.icmr.nic.in). Clinical genetics gained momentum as a consequence of pioneering efforts of senior geneticists like SS Agarwal, IC Verma, M Phadke, and IM Thomas. These services have progressively expanded over the last 30 years and as many as 40 trained Medical and Clinical geneticists practice all over the country at present. Many of the earlier geneticists have received part or whole of their training abroad, but the establishment of the Medical Genetics residency program as early as 27 years back at the Sanjay Gandhi Post Graduate Institute of Medical Sciences, Lucknow has been the primary training source for the newer generation of geneticists. Medical genetics services are presently available in 13 different premier cities of New Delhi, Lucknow, Vellore, Chandigarh, Hyderabad, Bangalore, Pune, Ahmedabad, Kochi, Trivandrum, Chennai, Manipal, and Mumbai as a separate department or units under the pediatrics department. In addition, many trained geneticists practice clinical genetics as part of their pediatric practice. The majority of the Medical Genetics outpatient services in the larger centers cater to an average of 20–40 patients on a daily basis. The cases include pediatric and adults, with antenatal patients comprising a significant proportion at many centers. Many geneticists and fetal ultrasonographers provide genetic sonogram and prenatal diagnosis services as part of their practice. Prenatal diagnosis has contributed to the popularity of medical genetics amongst obstetric practitioners. The growing significance of molecular genetics and cytogenetics in diagnosis as well as management of various cancers has also led to expansion of genetic diagnostic and counseling services in the country. As no trained genetic counselors are existent in the country, the medical geneticists also provide genetic counseling services to their patients. Hence, the work profile of a medical geneticist in India is varied and broad dependent upon the needs of the population, availability of other trained personnel and the kind of organization he/she is part of. Many of the geneticists take care of patients with dysmorphology, genetic metabolic disorders, disorders of sexual differentiation and development, neurodegenerative disorders, skeletal disorders, cancer genetics, adult onset disorders, etc. In addition, most medical geneticists are actively involved in diagnostic and research laboratory work, though some function primarily as clinical geneticists.

Diagnostic laboratory facilities are available both as part of an integrated medical genetics department/unit or as an independent facility. The integrated laboratories are supervised by laboratory geneticists or medical geneticists whereas the independent facilities are primarily supervised by laboratory geneticists. Cytogenetics, molecular genetics and biochemical genetics diagnostic tests are all available in most of these facilities. Some laboratory facilities have developed as part of other specialties like anatomy, biochemistry, hematology, and pathology in medical institutions. Recent years have seen marked expansion of private laboratories in the field of genetic diagnostic services. Availability of cytogenetic microarray and next generation based testing has brought the diagnostics to the state of art level but also needs responsible and knowledgeable geneticists for interpretation and to carry out the responsibility of appropriate pretest and post-test counseling. However, as is true for the clinical genetic services, most of the laboratory services are present in a few cities of the country in selective premier institutions. Patients from the rural and suburban areas of the country need to travel long distances to access both clinical and laboratory genetic services. The launch of the website <Geneticsindia> is an important milestone in the medical genetics in India. The website has been supported by Indian Council of Medical Research and is the result of council's funding support to many investigators working on various monogenic disorders. It provides a catalogue of genetic laboratories, tests and genetic counseling centers for use by clinicians as well as patients (http://www.geneticsindia.org).

There is no provision for genetic evaluation for the majority of the population and no national public health program is currently functional for carrier or newborn screening for genetic disorders. Population based carrier screening for beta thalassemia which was initiated as part of scientific projects is currently being started as government supported program in some states and has been recommended for countrywide implementation (Verma et al. 2011). Newborn screening has also been done on research basis and recently has commenced as a pilot program in one of the states (Kerala) of the country (Agarwal et al. 2015). Acceptance and performance of these programs and improved budgetary allocations may lead to possible expansion to nationwide screening in the new future. A birth defects registry has also been initiated in the last few years by a nongovernment organization but nationwide coverage has not been possible due to inadequate interinstitutional communication (http://www.fcrf.org.in/bdri_abus.asp).

Regulation of genetic diagnostic services is presently governed by the ICMR's ethics guidelines for biomedical research as no separate standards are available for the same. Prenatal diagnostic services are more stringently regulated by the Preconception and Prenatal Diagnostics Techniques (Prohibition of Sex Section) Act, 1994, which is primarily intended to curb the practice of prenatal sex determination for sex selection by female feticide (http://pndt.gov.in). This act outlines the requisite training of personnel and laboratory specifications for a genetics laboratory performing prenatal diagnosis. In addition this law also provides guidelines for a genetic counseling center and prenatal diagnosis facility; all of which form an important component of medical genetic services in India. However, separate laboratory standards are required for genetic diagnostic laboratories as presently most laboratories are following international guidelines or appropriate modifications by individual quality control standards. Another legislation which has implications for prenatal diagnosis practice in India is the MTP Act, 1974 (http://www.tcw.nic.in/Acts/MTP-Act-1971.pdf), which came into existence prior to the availability of prenatal testing with the primary aim to regulate illegal abortions. This act determines the upper limit of pregnancy termination as 20 weeks. This raises challenges for management and counseling of various late manifesting genetic syndromes and birth defects, and has led to proposal of an amendment recently (Phadke et al. 2011a).

## Spectrum of Genetic Disorders

Almost all known reported genetic disorders are found in India. Medical geneticists as well as clinicians from other fields have been reporting such cases in the medical literature since their delineation. However, the data has been from limited institutions or individuals and nationwide prevalence of most disorders is not known. Beta thalassemia is the most common genetic disorder and has a carrier frequency of 5–17% with some population groups having even higher carrier rates (Colah et al. 2008). Most of the genetic centers in India initiated their molecular diagnostic laboratories by establishing genetic testing of beta thalassemia and data of mutation spectrum from various states and population groups is available (Varawalla et al. 1991; Agarwal et al. 2000; Das et al. 2000; Colah et al. 2009; Sinha et al. 2011). The pan-ethnic genetic conditions like Spinal Muscular Atrophy and Duchenne Muscular Dystrophy have prevalence and carrier rates similar to worldwide prevalence. Common genetic disorders like Hemophilia, Achondroplasia, Huntington disease, Lysosomal storage disorders, and many others have been reported in large numbers from all parts of the country. Information from small scale newborn screening projects, case reports and personal experiences of clinicians reveals that all inborn errors of metabolism are seen in India (Ambani et al. 1979; Devi et al. 1993; Verma 2000; Verma and Bijarnia 2002; Sahai et al. 2011; Lodh and Kerketta 2013). Dysmorphology diagnosis has been of interest to many pediatricians as is reflected by a number of case reports of rare syndromes and new manifestations of known syndromes in pediatrics journals of India (Nadkarni and Nadkarni 1964; Shah 1965; Chaudhuri and Chaudhuri 1966; Agarwal et al. 1974; Menon et al. 1980; Mehta and Agarwal 1981; Pai 1986; Phadke et al. 1986; Deka et al. 1991; Kher et al. 1994; Phadke 2011cc). Documentation of facial phenotypes of Indian patients has been of interest to clinicians across the world (Patil et al. 2012). Many new syndromes have been reported by Indian authors and have been included in OMIM and the London Dysmorphology Database (Verma et al. 1975; Agarwal et al. 1994; Sharma et al. 1994a; Phadke et al. 1998, 1999, 2003, 2006, 2011c; Pradhan et al. 1999; Panigrahi et al. 2002; Puri and Phadke 2003; Parmar and Muranjan 2004; Ghosh et al. 2007; Phadke and Dalal 2007; Baskar et al.^2009^; Girisha et al. 2010) (Table1). However, due to cases limited to nuclear families the traditional linkage methods have not been useful in identifying causative genes. With the genomic tools now available, these cases, especially from consanguineous families, are a good candidates for gene mapping (Geetha et al. 2014). The high rates of consanguinity and practice of endogamy has been thought to lead to the high prevalence of rare recessive conditions as well as unique founder mutations in specific population groups. Although there is no data to prove population level high prevalence of autosomal recessive disorders in populations with high consanguinity our [S.R. Phadke, unpubl. data] shows that consanguinity is 2–3 times more common in families with rare autosomal recessive disorders (26.6%) as compared to the most common autosomal recessive disorder namely; beta thalassemia (10.7%). Use of SNP microarray to identify regions of homozygosity and candidate genes in these regions is a strategy which can be successfully used in the consanguineous families (Stephen et al. 2015). Some conditions found to be relatively common have been Van Der Knaap disease with a founder mutation in the Agarwal community from north western India, calpainopahy, recessive forms of Osteogenesis imperfecta, Progressive pseudorheumatoid arthropthy of childhood and Handigodu disease from a specific community in South India (Gorospe et al. 2004; Pathak et al. 2010; Sachdeva et al. 2011, 2012; Shukla et al. 2011; Bashyam et al. 2012; Dalal et al. 2012; Bidchol et al. 2014; Ankala et al. 2015). However, the available data on monogenic malformation syndromes and metabolic disorders from India though extensive, represents only tip of the iceberg as a large population still does not have access to the clinical genetics services due to cost and limited number of genetic centers.

**Table 1:** Some novel genetic syndromes reported from India

Name of syndrome	OMIM ID	Reference
Short rib polydactyly syndrome (SRD) type III (Verma-Naumoff syndrome)	263510	Verma et al. (1975)
Handigodu Disease	613343	Agarwal et al. (1994)
Camptosypolydactyly, complex disorganization type	^*^607539	Phadke et al. (2003)
Mental retardation, ptosis and polydactyly	–	Panigrahi et al. (2002)
Microcephaly, micropenis	–	Pradhan et al. (1999)
Van Den Ende Gupta syndrome of blepharophimosis, contractual arachnodactyly and characteristic facies	–	Phadke et al. (1998)
Preaxial brachydactyly with abduction of thumbs and hallux varus	–	Sharma et al. (1994a)
A newly recognized syndrome with double upper and lower lip, hypertelorism, eyelid ptosis, blepharophimosis, and third finger clinodactyly	–	Parmar and Muranjan (2004)
Handless footless syndrome	–	Phadke et al. (2006)
Short stature, ulnar deviation of hands with absent carpals and joint contractures	–	Phadke and Dalal (2007)

Chromosomal aneuploidies and common microdeletion syndromes have also been found in large numbers. In recent years, with availability of chromosomal microarray platforms, rarer microdeletion/duplication syndromes are also being detected (Gupta et al. 2014). Due to cost limitations, many centers use multiplex ligation dependent probe amplification assay for diagnosis of subtelomeric and common microdeletion/duplication syndromes (Boggula et al. 2014).

Birth defects are common in the Indian population, with various environmental factors like nutritional deficiencies contributing in addition to genetic predispositions (Agarwal et al. 1991; Sharma et al. 1994b; Bhat and Babu 1998; Suresh et al. 2005). The involvement of Medical geneticists in antenatal imaging as well as perinatal autopsies has led to recognition of known as well as novel malformation syndromes in many such cases (Sankar and Phadke 2006).

Familial cancers and cancer prone monogenic syndromes have been also an important part of referral to a clinical genetics center. Advanced Centre for Treatment, Research and Education in Cancer in Mumbai has the largest cancer genetics clinic and provides molecular diagnosis for many monogenic cancer syndromes. Many other centers have research and clinical services in the field of cancer genetics (Kotnis et al. 2005; Sudhakar et al. 2007; India Project Team of the International Cancer Genome Consortium 2013; Gokhale et al. 2014; Wasson et al. 2014). Genetic studies and research in ophthalmological disorders by many renowned eye centers in south India have been remarkable (Gandra et al. 2008; Singh et al. 2009 Kannabiran et al. 2012).

## Academic Programs in Medical Genetics

Training programs in Medical Genetics are available currently in four centers in the country. Of these a 3 year residency program at Sanjay Gandhi Post Graduate Institute of Medical Sciences (SGPGIMS), Lucknow has been the oldest one currently running in its 28th year and has the distinction of being the only such program in an academic institution under the Government of India. Other programs are shorter courses of 1–2 years duration in private institutions. The Department of Medical Genetics at SGPGIMS, Lucknow also holds a 2 week long program every year for clinicians. It is aimed at training clinicians, medical college teachers, postgraduate students, and private practitioners in basic genetics an approach to common genetic problem using diagnostics and genetic counseling. This is a very popular program and is supported by Indian Council of Medical Research for more than 10 years. Many other centers have been organizing seminars and workshops in genetics and most of the medical conferences have improved the coverage of genetics related topics in their scientific programs. Medical Council of India has been taking actions to upgrade genetics curricula in undergraduate and postgraduate medical courses. Besides these, there have been in recent years commencement of 1.5 year programs for laboratory geneticists at the Centre for DNA Fingerprinting and Diagnostics, Hyderabad under the aegis of Society for Indian Academy of Medical Genetics. A masters course in genetic counseling started recently in Medical Genetics department of Kasturba Medical College, Manipal is the first step in creating much needed trained genetic counselors trained in clinical settings. Shortly, a 3 year residency program under the National Board of Examinations is scheduled to start in three centers in different parts of the country. The Society for Indian Academy of Medical Genetics has come into existence since the year 2012 <iamg.in> and has brought together the Medical and Clinical geneticists across the country. The academy has been conducting monthly teleconferences furthering exchange of interesting clinical and research data among different centers. The academy also has a quarterly publication Genetic Clinics where interesting cases and latest advances in the field are published with an aim to reach out to doctors from other fields (www.iamg.in). The Indian Society of Human Genetics has also been active in popularizing basic scientific research in genetics through its yearly meetings and an indexed journal, the Indian Journal of Human Genetics (www.ijhg.com).

## Research

Various institutions with integrated medical genetics centers as well as institutions committed to scientific research have been instrumental in pioneering genetic and genomic research in India. The research pertaining to monogenic disorders has been under the purview of primarily medical geneticists whereas research related to cancer genetics, multifactorial disorders and population genetics has been carried out primarily by nonmedical scientists. The research is funded by individual institutions as intramural grants or by national organizations like the Indian Council of Medical Research, Department of Biotechnology, Department of Science and Technology and Council for Scientific and Industrial Research. The premier institutions committed to scientific research in the field of genetics have been the Centre for Cellular & molecular Biology at Hyderabad, National Institute of Biomedical Genomics at Kalyani, Advanced Centre for Treatment, Research and Education in Cancer at Mumbai, Centre for DNA Fingerprinting and Diagnostics at Hyderabad, Institute of Genomics and Integrative Biology at New Delhi and Indian Institute of Science at Bangalore. The research work from these centers have provided insights into the genetic landscape of the Indian population, the polymorphic markers defining different population groups, multifactorial disease association, genes predisposing to sporadic cancers prevalent in the country like oral & cervical cancers, genetic markers governing infectious disease susceptibility, genes responsible for inherited cardiomyopathies, etc. (Palanichamy et al. 2004; Dhandapany et al. 2009; Ronsard et al. 2014; Sharma et al. 2014; Singh et al. 2014; Talwar et al. 2014; Walia et al. 2014). Pharmacogenomics is also an important area of work of many scientists (Senapati et al. 2014). However, the contribution of Indian scientists to gene mapping has been limited. Sequencing of the first Indian genome and establishment of the Indian Genome Variation Consortium have been notable landmarks achieved by these scientific groups which are likely to further genetic research in the field of both complex and Mendelian genetics (Narang et al. 2010). Besides playing pivotal role in scientific research, many national funding organizations have also been instrumental in the establishment of DNA based diagnostics and medical genetics centers since last three decades. Research work by the medical geneticists has encompassed establishing mutation profiles of well-defined genetic disorders, mapping of genes for novel syndromes, cytogenetic delineation of genomic disorders, molecular etiologies of birth defects and other fetal syndromes, and various other research studies primarily targeted to single gene and chromosomal disorders aimed at immediate translation into patient care. Recent years have seen publication of genetic and mutation profiles for large case series of osteogenesis imperfecta, skeletal dysplasias, and lysosomal storage disorders. These studies have revealed the unique molecular profile of patients from the India with identification of many novel mutations not previously reported in other populations (Gorospe et al. 2004; Pathak et al. 2010; Sachdeva et al. 2011, 2012; Shukla et al. 2011; Bashyam et al. 2012; Dalal et al. 2012; Mistri et al. 2012; Bidchol et al. 2014; Ankala et al. 2015; Stephen et al. 2015; A. Uttarilli, P. Ranganath, S.J.M. Nurul Jain, K.P. Chintakindi, A. Sinha, I.C. Verma, unpubl. data) (Table2). In recent times, Medical geneticists have started using exome sequencing in investigation of unidentified genetic disorders (Shah et al. 2014; A.B. Dalal, A.D. Bhowmik, D. Agarwal, S.R. Phadke, unpubl. data).

**Table 2:** Mutation profile of some common genetic disorders from India

Disease (*Gene*)	Common mutations in India (%)		Worldwide common mutations		Reference
Beta thalssemia (*HBB*)	North India	IVS 1–5 G>C (44.8%) 619 bp deletion (13%) Codon 8/9 +G (11%) Codon 41/42 -TCTT(10.3%) IVS 1–1 G>T (8.6%)	Mediterranean	-87C>G IVS1-1G>A IVS1-6T>C IVS1-110G>A cd39C>T	Varawalla et al. (1991), Das et al. (2000), Agarwal et al. (2000), Colah et al. (2009), Sinha et al. (2011), Cao et al. (2000),
	Central India	IVS 1-5 G>C (49.8%) 619 bp deletion (16.6%) IVS 1-1 G>T (8.9%) Codon 8/9 +G (7.3%)	Middle east	cd8-AA cd8/9+G IVS1-5G>C cd39C>T	
	West India	IVS 1-5 G>C (50.7%) 619 bp deletion (14.2%) IVS 1-1 G>T (8.7%) Codon 15 G>A (7.6%)	Chinese	-28A>G 17A>T 41/42-TTCT	
	East India	IVS 1-5 G>C (71.6%) Codon 41/42 -TCTT(6.1%) Codon 15 G>A (5.6%)	Thai	-28A>G 17A>T 19A>G	
	South India	IVS 1-5 G>C (68%) Codon 15 G>A (8.8%) Poly A site T>C (4.7%)	African/American African	-88C>T -29A>G IVS1-5G>T cd24T>A	
Cystic fibrosis (*CFTR*)	ΔF508 deletion (31-34%) p.R1162X (2.2%) p.M1T (0.8%) S559N (0.8%)		Northern European heritage ΔF508 (66%) G542^*^ (2.4%) G551D (1.6%) N1303Lys (1.3%) W1282^*^ (1.2%)		Moskowitz et al. (2001), Sachdeva et al. (2011, 2012)
Hypohidrotic/Anhidrotic ectodermal dysplasia (*EDAR & EDA*)	*EDAR* mutations- 46% c.1144G>A in 5/12 families with *EDAR* mutations *EDA* mutations- 42%		EDAR - 15–20% EDA - 55–60%		Bashyam et al. (2012), Wright et al. (2003),
Metachromatic leukodystrophy (*ARSA*)	c.459+1G>A- 2/16 families		European population c.459+1G>A (15–28%) p.Pro426Leu (15–27%) p.Ile179Ser (2–13%) Japanese population p.Gly99Asp (45%)		Shukla et al. (2011), Fluharty (2006),
Morquio syndrome A (*GALNS*)	p.Ser287Leu (8.82%) p.Phe216Ser (7.35%) p.Asn32Thr (6.61%) p.Ala291Ser (5.88%)		Latin American p.Arg386Cys Irish, Australian p.Ile113Phe p.Thr312Ser Colombia p.Gly301Cys		Bidchol et al. (2014), Regier et al. (2013),
Progressive Pseudorheumatoid arthropathy of childhood (*WISP3*)	c.1010G>A – 10/25 families c.233G>A- 4/25 families		Scarce data c.156C>A- Turkey, Lebanon, Syria c.327C > A- Turkey c.727_731delGAGAA- Turkey		Dalal et al. (2012), Garcia Segarra et al. (2012)
Megalencephalic leukodystrophy with subcortical cysts (*MLC1*)	320insC – 31/31patients from Agarwal community		Libyan and Turkish Jews c.176G>A Japanese c.278C>T		Gorospe et al. (2004), van der Knaap and Scheper (2003)
Tay Sachs disease (*HEXA*)	c.1385A>T (p.E462V)-6/15 families from state of Gujarat		Ashkenazi Jews c.1274_1277dupTATC c.1421+1G>C French Canadian 7.6-kb del		Kaback and Desnick (1999), Mistri et al. (2012)
Mucopolysaachridosis type VI (*ARSB*)	p.W450C (c.1350G>C)- 4/14 families, Founder mutation p.L98R (c.293T>G),- 3/14 families, Hot spot mutation		p.Y210C p.R152W- 50% in some European nations		A. Uttarilli, P. Ranganath, S.J.M. Nurul Jain, K.P. Chintakindi, A. Sinha, I.C. Verma, unpubl. data, Karageorgos et al. (2007)

## Patient Support Groups

Patient support groups play a very important role in the care of patients with genetic disorders in India where almost no support exists in the public health care system for these individually rare and mostly unrecognized conditions (http://downsyndrome.in, http://www.mdindia.org). Many of these conditions require life-long therapy and support, remaining potentially incurable. Others like lysosomal storage disorders and various inborn errors of metabolism have highly expensive treatment modalities. The support groups for hemophilia and thalassemia patients have been in active existence since many years and have been instrumental in state sponsored treatment for these disorders in many regions of India (http://www.thalassemicsindia.org, http://www.hemophilia.in). Patient support groups for Rett syndrome and lysosomal storage disorders are gaining roots in the country and have been working toward creating awareness and advocacy for subsidized therapy and state sponsored support for these rare conditions (http://www.rettsyndrome.in, http://www.lsdss.org). Organization for rare diseases has recently been established (http://ordindia.org, Rajasimha et al. 2014). Along with facilitating availability of medical facilities, the patient support groups' activities are surely bringing hope for these patients and their families and are ensuring that they receive appropriate attention from the policy makers.

## Conclusion and Challenges Ahead

Though this article does not claim to represent an all inclusive account of the past and present of medical genetics in India; it is an attempt to revisit the past developments and assess the current scenario. The progress over last 2–3 decades have brought state of art molecular diagnostics to India and along with excellent clinical skills in dysmorphology and metabolic disorders; specialty of medical genetics has been well-established in India bringing the greatest benefit to the patients with genetic disorders and their families. However, though a beginning has been made, the research in the area of genetic disorders still lags behind in the areas of understanding pathogenesis and new drug developments. Research in gene therapy and stem cell therapy is almost nonexistent. Research and development in these areas and recombinant products will go a long way in making the new therapies available to Indian patients at affordable costs.

The complex genetic architecture, the vast numbers, the high rates of consanguinity, all position India as a Pandora's Box in the field of genetics. A large volume of clinical material remains unutilized for research. Despite ongoing promising expansions in the field and availability of state of the art clinical and diagnostic facilities; the number of geneticists and laboratories are too scarce to cater to the huge population of India. Improved health budget allocations, sensitization of the health authorities and policy makers toward the burden of genetic diseases, inclusion of genetics in medical curriculum and fostering awareness amongst medical practitioners from other fields are important steps required for this purpose. Screening programs like newborn screening, thalassemia prevention and Down syndrome screening need to be implemented by government rather than current pattern of private providers. Data regarding prevalence of rare diseases, especially treatable ones and their registries is essential for empowerment of patient advocacy group and proactive involvement of policy makers in implementation of appropriate screening and management programs for such patients. Medical Council of India needs to take up the education of modern genetics at all levels of medical curricula; so that the coming generation of medical practitioners is prepared for the era of molecular medicine. There is a need for improved collaboration between clinical geneticists and basic scientists so that the extensive clinical material can be used research purposes. Despite numerous reports of new syndromes from India, there has been no impact in the area of gene mapping. Our unpublished experience shows high rates of consanguinity in families with rare genetic disorders, which are mines for genehunting. Recent technologies of exome sequencing in combination with SNP microarray can identify causative gene in very small families as well (Shah et al. 2014, A.B. Dalal et al. unpubl. data). With the tremendous speed of gene mapping achieved by exome sequencing, the world is expecting to identify causative genes for all monogenic disorders and India should be able to contribute to this last lap of gene mapping marathon.
